# Splicing defect and functional characterization of the *ETFDH* c.1049G > A VUS underlying transient MADD: an iPSC and minigene study

**DOI:** 10.1186/s13023-026-04407-1

**Published:** 2026-05-29

**Authors:** Rui Dong, Xiaochen Wang, Haiyan Zhang, Guohua Liu

**Affiliations:** 1 Pediatric Research Institute, Children’s Hospital Affiliated to Shandong University (Jinan Children’s Hospital), Jinan, China; 2 Shandong Provincial Clinical Research Center for Children’s Health and Disease, Jinan, China

**Keywords:** *ETFDH*, Variants of uncertain significance (VUS), Induced pluripotent stem cells (iPSCs), Minigene

## Abstract

**Background:**

Interpreting variants of uncertain significance (VUS) is challenging in patients with atypical or transient metabolic presentations.

**Methods:**

We combined induced pluripotent stem cell (iPSC) models and minigene assays to characterize an *ETFDH* VUS (c.1049G > A) in a neonate who presented with acute metabolic decompensation characterized by fatty acid oxidation defects but showed normalized metabolic profiles by 14 months of age.

**Results:**

Patient-derived iPSCs and minigene assays confirmed that c.1049G > A induces predominant exon 9 skipping, resulting in an in-frame 48-amino acid deletion within the FAD-binding domain. Structural modeling based on the human ETFDH crystal structure revealed that this deletion disrupts the core architecture of the FAD-binding domain, notably by eliminating the critical stabilizing interaction between Arg364 and Glu246, predicting compromised FAD binding affinity. Consistent with this structural defect, Western blot analysis showed markedly reduced ETFDH protein levels (< 10%) in patient cells versus heterozygous parents (~ 40%). Collectively, these data indicate that while the variant causes substantial molecular impairment explaining the neonatal crisis, the presence of residual protein likely supports metabolic homeostasis under non-stressful conditions.

**Conclusions:**

The c.1049G > A variant causes significant splicing disruption and partial protein loss, explaining the transient nature of the neonatal decompensation rather than a classic, persistent MADD phenotype. Furthermore, this study underscores the utility of iPSC models in resolving VUS cases where clinical biochemistry normalizes over time, bridging the gap between ambiguous genetic data and precise clinical management.

**Supplementary Information:**

The online version contains supplementary material available at 10.1186/s13023-026-04407-1.

## Introduction

 Defects in electron transfer flavoprotein dehydrogenase (*ETFDH*) cause multiple acyl-CoA dehydrogenase deficiency (MADD; MIM #231680), a rare autosomal recessive disorder impairing fatty acid β-oxidation and amino acid metabolism [1, 2]. MADD exhibits a broad clinical spectrum, ranging from severe neonatal forms with congenital anomalies (Types I and II) to mild late-onset presentations (Type III) [3, 4]. Clinical manifestations vary from life-threatening hypoglycemia, hypotonia, and cardiomyopathy to recurrent rhabdomyolysis and lipid storage myopathy, with polycystic kidney disease increasingly recognized as a specific phenotype of early-onset MADD [5–8]. Diagnosis relies on characteristic metabolic profiles—elevated multiple acylcarnitines in blood and increased urinary excretion of glutaric, ethylmalonic, and dicarboxylic acids—confirmed by biallelic pathogenic *ETFDH* variants [9, 10]. However, these biochemical signatures can be transient or atypical in mild cases, often evading detection and complicating diagnosis.

A distinct genotype-phenotype correlation exists: missense mutations often retain residual enzymatic activity, correlating with riboflavin-responsive, late-onset phenotypes, whereas splicing, nonsense, and frameshift mutations typically cause complete loss of function, leading to severe early-onset MADD [11, 12]. While this framework guides initial clinical expectations, significant heterogeneity remains, particularly when interpreting variants of uncertain significance (VUS) in compound heterozygosity with atypical or evolving clinical presentations.

In this study, we identified compound heterozygous *ETFDH* variants—a novel VUS (c.1049G > A) and a known pathogenic allele—in a neonate presenting with atypical metabolic decompensation that spontaneously normalized by 14 months of age. To elucidate the molecular impact of c.1049G > A, we employed patient- and parental-derived induced pluripotent stem cells (iPSCs) for splicing analysis and protein quantification, complemented by minigene assays. By linking the confirmed splicing defect to residual protein levels, we clarify how this specific molecular impairment underlies the transient clinical phenotype. This approach not only provides a precise paradigm for characterizing splicing defects in uncertain variants but also underscores the critical utility of iPSC models in diagnosing metabolic disorders with complex, evolving clinical trajectories.

## Methods

### Study participants and samples

A female pediatric proband with suspected fatty acid metabolism disorder and her parents were recruited from the Children’s Hospital Affiliated to Shandong University. Clinical data and peripheral blood samples were collected from the trio for phenotypic characterization and genetic analysis.

### Genetic analysis and bioinformatics pipeline

Genomic DNA was isolated (QIAamp DNA Blood Midi Kit) and analyzed by whole-exome sequencing (WES) (Illumina Novaseq 6000; 150x depth). Variants were annotated (hg19) with Sentieon, filtered per ACMG guidelines [13], and validated by Sanger sequencing (primers 1049 and 1227; Table S1). Splice impact of c.1049G > A was predicted with SpliceAI and Alamut. Structural modeling (ETFDH) was performed using SWISS-MODEL and PyMOL.

### iPSC generation and validation

Peripheral blood mononuclear cells (PBMCs) were reprogrammed into iPSCs using episomal plasmids encoding OCT4, SOX2, KLF4, BCL-XL, and c-MYC through electroporation (Nucleofector system, Lonza). The generated iPSCs were rigorously validated for pluripotency via immunofluorescence and RT-PCR analysis. Their tri-lineage differentiation potential was further confirmed by an in vitro embryoid body (EB) formation assay, followed by qRT-PCR quantification of lineage-specific markers for ectoderm, mesoderm, and endoderm. Genetic stability was assessed by karyotyping, and the retention of specific genetic variants was verified using Sanger sequencing. The primers used in the experiments are listed in Table S2.

### RNA and protein analysis

RNA was extracted (TRIzol), reverse-transcribed (Takara PrimeScript™), and amplified by nested PCR (primers E1 and E2; Table S1). *ETFDH* splicing isoforms were analyzed via RT-PCR and Sanger sequencing. Cells were lysed, and proteins separated on 10% SDS-PAGE gels, transferred to PVDF membranes, and blocked with 5% skim milk. Membranes were incubated overnight with anti-ETFDH antibody (Proteintech; raised against aa 467–665) or anti-GAPDH antibody, followed by HRP-conjugated secondary antibodies. Signals were detected using ChemiScope 5300 (Clinx).

### Minigene splicing assay

To evaluate the pathogenicity of c.1049G > A, a minigene containing exon 8 (141 bp)-intron 8 (1,287 bp)-exon 9 (144 bp)-partial intron 9 (518 bp) was constructed in pcMINI-N (MCS-IntronB-ExonB backbone). The genomic fragment was amplified from normal gDNA via nested PCR using two primer pairs (A and B; Table S1), cloned into the vector, and validated by sequencing. Mutant constructs were generated by site-directed mutagenesis (primers pcMINI and MT) and confirmed by sequencing. Recombinant plasmids were transfected into HeLa and HEK293T cells (Lipofectamine 2000). After 48 h, RNA was isolated, reverse-transcribed, and amplified by RT-PCR (primers N). Splicing isoforms were analyzed via agarose gel electrophoresis and sequencing.

### Statistical analysis

Quantitative data from qRT-PCR and Western blotting were obtained from at least three independent biological replicates and are presented as the mean ± SD. Statistical differences between two groups were assessed using the unpaired two-tailed Student’s t-test, and comparisons among multiple groups were performed using one-way ANOVA followed by Tukey’s post-hoc test. All analyses were conducted using GraphPad Prism 9.0. A p-value < 0.05 was considered statistically significant (*p < 0.05, **p < 0.01, ***p < 0.001, ****p < 0.0001).

## Results

### Clinical presentation and initial management

A female neonate, born at 35 + 1 weeks via cesarean section due to maternal complications, presented with immediate cyanosis and severe respiratory distress complicated by pneumothorax. Despite initial resuscitation, she rapidly progressed to persistent hypoxemia, shock, and multi-organ dysfunction within 24 h, necessitating NICU admission. Clinical examination revealed fever, hemodynamic instability, generalized hypotonia, and depressed neurological reflexes, without dysmorphic features. Laboratory evaluation confirmed systemic inflammation, coagulopathy, and significant metabolic derangements (detailed clinical characteristics and laboratory values are provided in Table S3). Notably, acute-phase metabolic screening identified elevated butyrylcarnitine (C4) in blood and increased 4-hydroxyphenyllactic acid (4-HPLA) in urine (Table 1). Imaging further identified intracranial hemorrhage, hydrocephalus, and persistent pulmonary hypertension (PPHN). Management comprised respiratory and hemodynamic support, antibiotics, and specific metabolic therapy with L-carnitine. The patient responded to treatment and was discharged after one month. At the 14-month follow-up, while the patient remained clinically stable, notably, both blood and urine metabolic profiles had returned to normal ranges, suggesting a transient metabolic disturbance.

**Table 1:** Results of metabolic profiling

Metabolites	Day 8	Day 24	14 months of age
Blood mass spectrometry profile			
C0-Free carmitine (reference, 7-51.4 umol/L)	7.4	119.19↑	19.38
C2-Acetylcanitine (reference, 9–50 umol/L)	6.52↓	39.64	10.8
C3-Propionylcarnitine (reference, 0.5–4.7 umol/L)	1.87	2.3	1.48
C3/C0 (reference, 0.01–0.2 umol/L)	0.25↑	0.02	0.08
C4-Butyrylcarnitine (reference, 0.05–0.53 umol/L)	0.23	0.82↑	0.25
C4/C2 (reference, 0-0.03 umol/L)	0.04↑	0.02	0.02
C5-Isovaleryl-L-Carnitine (reference, 0.04–0.42 umol/L)	0.1	0.3	0.12
C8-Octanoylcarnitine (reference, 0.02–0.20 umol/L)	0.04	0.13	0.05
C14:1-Tetradecenoyicarnitine (reference, 0.01–0.30 umol/L)	0.04	0.03	0.02
C16-Hexadecanoylcanitine (reference, 0.44–6.31 umol/L)	1.17	1.09	0.97
C18-Octadecanylcanitine (reference, 0.16–1.85 umol/L)	0.26	0.42	0.35
Urine mass spectrometry profile			
4-hydroxyphenyllactic acid (reference, 0–2 mmol/L)	129.7↑	-	0

Note: “–” = not detected

### Genetic findings

**Fig. 1:**
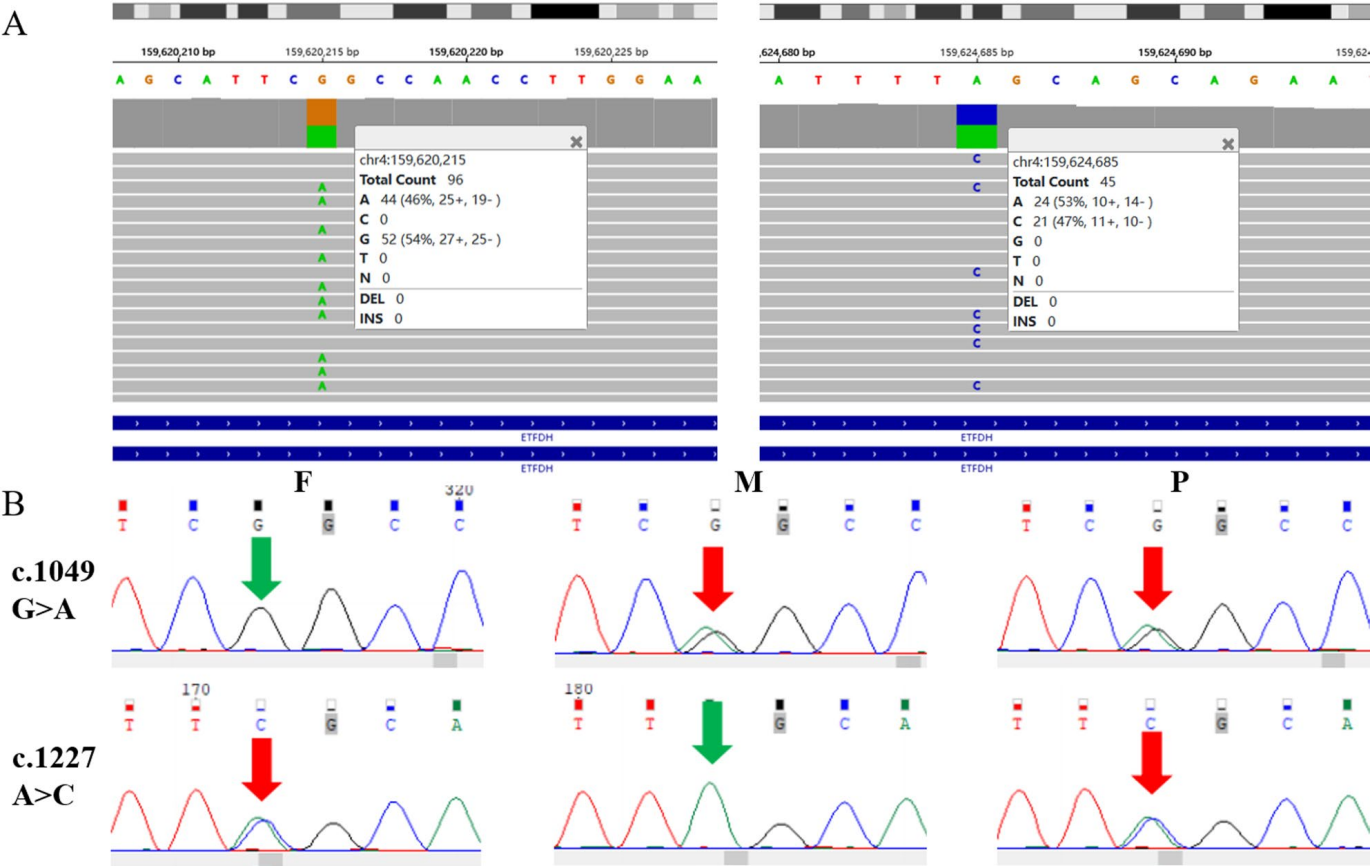
WES and Sanger sequencing revealed the paternally-inherited variant c.1227 A > C (p.Leu409Phe) and the maternally-inherited variant c.1049G > A (p.Arg350Gln)

WES identified heterozygous variants c.1049G > A (p.Arg350Gln) and c.1227 A > C (p.Leu409Phe) in the *ETFDH* gene (NM_004453.2) of the patient. Sanger sequencing confirmed the presence of both variants and established their compound heterozygous inheritance (Fig. 1). The c.1227 A > C variant, a known pathogenic variant cataloged in the HGMD, has been recurrently reported in MADD patients worldwide and represents a common variant hotspot in Chinese populations, observed in both homozygous and compound heterozygous states [14–17]. The c.1049G > A variant is annotated in ClinVar with conflicting pathogenicity classifications and lacks prior literature reports. Computational predictions were discordant: SpliceAI predicted no splicing impact (score < 0.1), while Alamut suggested potential splice modulation. Despite a low pathogenicity likelihood score (Varsome: 0.17), its low minor allele frequency (gnomAD: 0.00017; PM2) and occurrence in trans with a pathogenic variant (PM3) supported a potential contributory role in a compound heterozygous context. Per ACMG/AMP guidelines, this variant was classified as a VUS.

### Generation and functional validation of patient-derived iPSCs

**Fig. 2:**
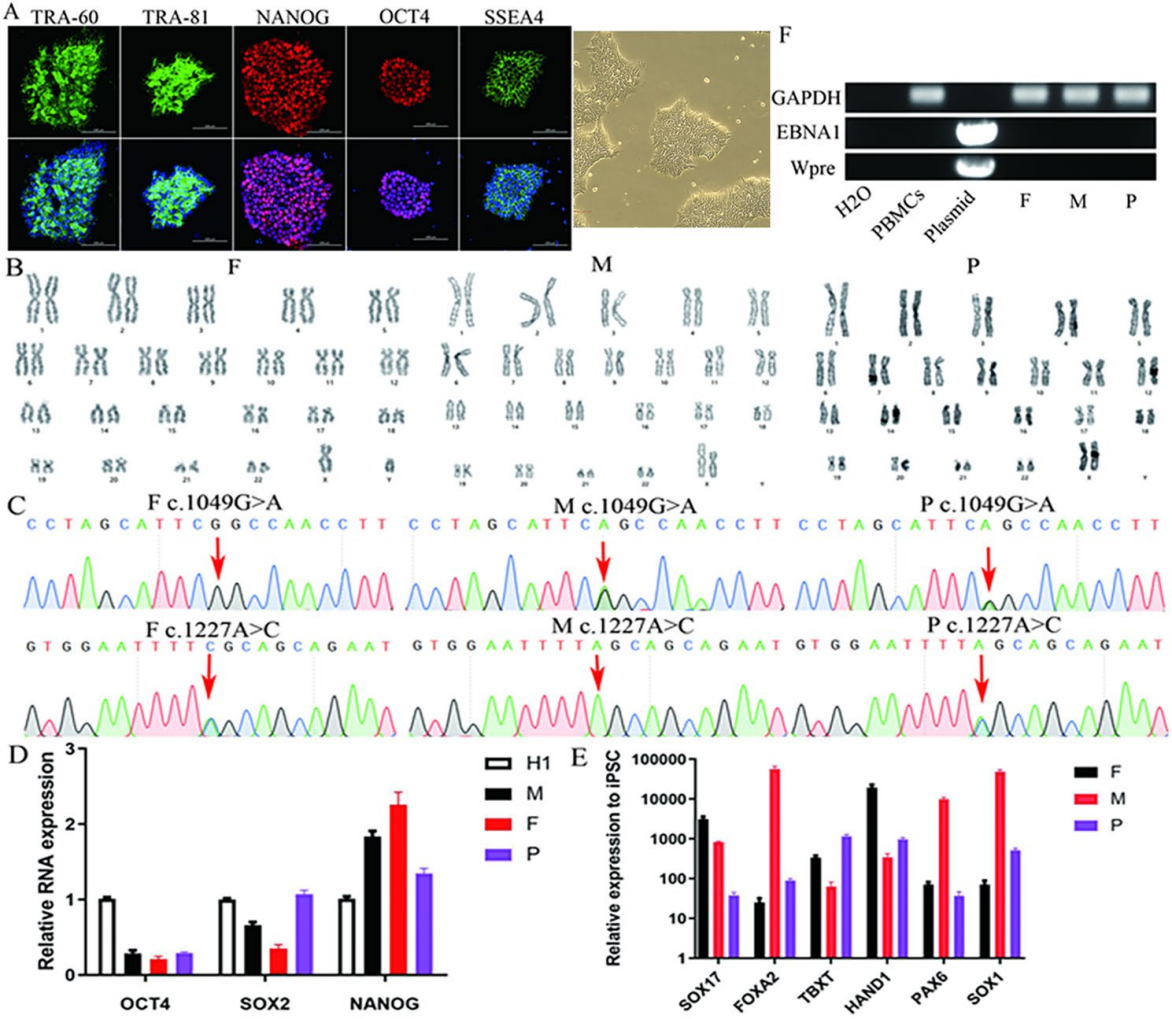
Characterization of iPSCs. (**A**) Immunofluorescence analysis of pluripotency markers and representative phase-contrast images of the generated iPSCs. Scale bar 100 μm. (**B**) The karyotype analysis of the iPSCs. (**C**) Sanger sequencing of variants on *ETFDH* in iPSCs. (**D**) qTR-PCR analysis of OCT4, SOX2 and NANOG in iPSCs. (**E**) qRT-PCR analysis of lineage-specific markers (PAX6, SOX1 for ectoderm; TBXT, HAND1 for mesoderm; SOX17, FOXA2 for endoderm) in differentiated embryoid bodies (EBs) derived from the iPSCs

All iPSC lines derived from the patient and their parents exhibited hallmark pluripotency features, including robust expression of pluripotency markers (e.g., OCT4, NANOG), maintenance of a normal karyotype, and trilineage differentiation capacity in vitro (Fig. 2A, B, D). Targeted sequencing confirmed retention of the *ETFDH* c.1049G > A variant in patient- and maternal-derived iPSCs, recapitulating the genetic profile of the original PBMCs (Fig. 2C).

### Splicing modulation by the c.1049G > a variant

**Fig. 3:**
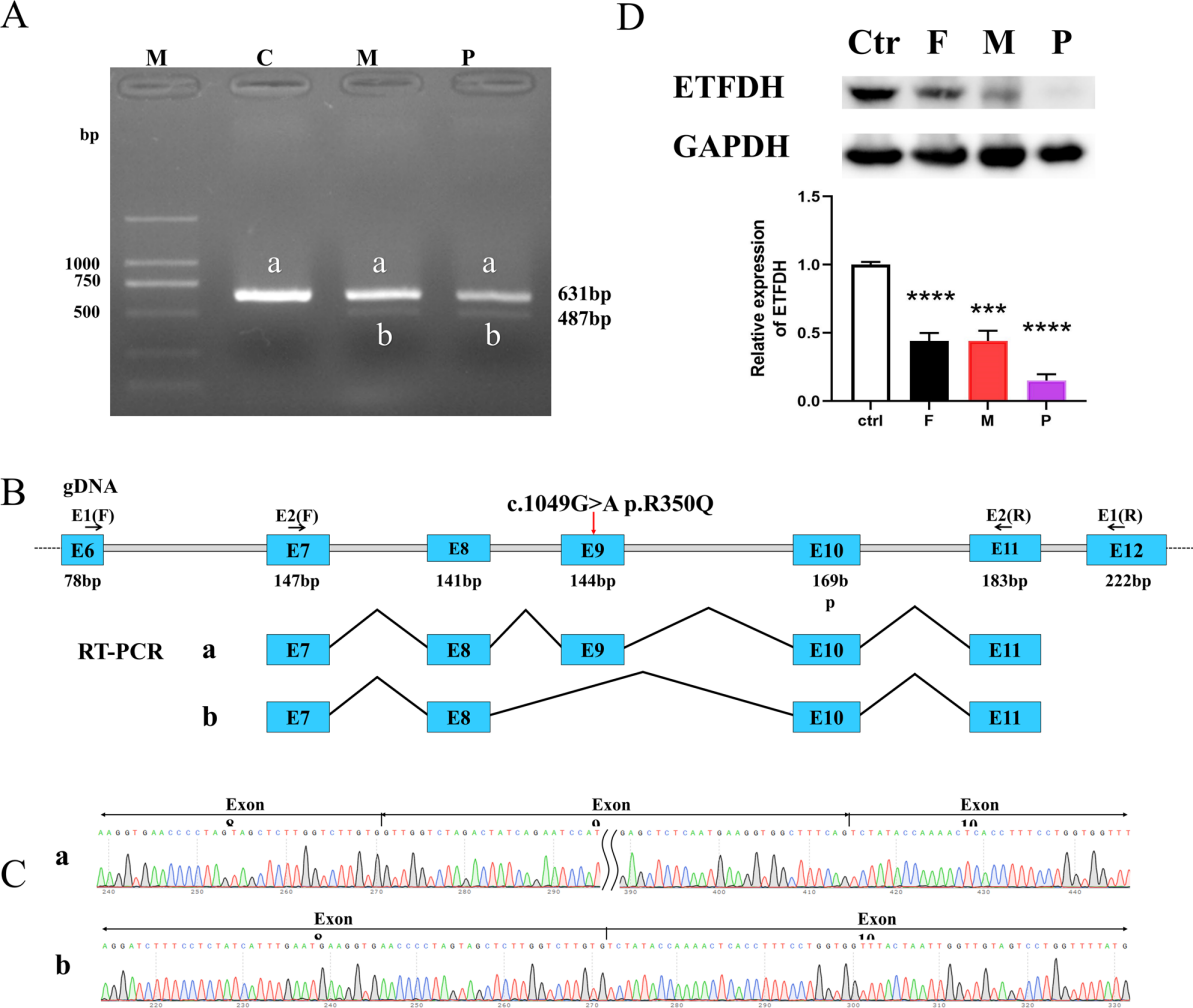
Analysis of Alternative Splicing Patterns. (**A**) Agarose gel electrophoresis of RT-PCR products. Band a: control; Bands a and b: patient and mother samples. (**B**) Schematic representation of primer design and splice variant depiction. The red arrow indicates the variant location. (**C**) Sequencing chromatograms for Bands a and b, showing splice variant sequences. (**D**) Western blot analysis of ETFDH protein expression in iPSCs from control, patient, father, and mother. Relative expression compared to control. ***P < 0.005, **** *P* < 0.0001

Transcript analysis of patient- and maternal-derived iPSCs identified two isoforms: a wild-type transcript and an exon 9-skipped isoform (Fig. 3A–C). Although the exon 9-skipped isoform is annotated in Ensembl, its absence in healthy controls (Fig. 3A) indicates that the c.1049G > A variant promotes aberrant usage of this cryptic splice event, leading to a reduction in functional transcripts. Reduced wild-type transcript abundance in the patient (vs. controls and carrier mother) was corroborated by agarose gel band intensity (Fig. 3A), suggesting variant-mediated suppression of canonical splicing.

### ETFDH protein deficiency in patient-derived cells

Western blot analysis of iPSC-derived models showed a marked reduction in ETFDH protein levels: patient samples exhibited < 10% of wild-type expression, while parental carrier samples retained 40% of control levels (Fig. 3D). This dose-dependent decrease aligns with the compound heterozygous genotype. Critically, the detection of residual ETFDH protein (< 10%) in patient cells, albeit at significantly reduced levels compared to carriers, suggests a partial loss-of-function mechanism rather than a complete null state.

### Minigene validation of splicing dysregulation

**Fig. 4:**
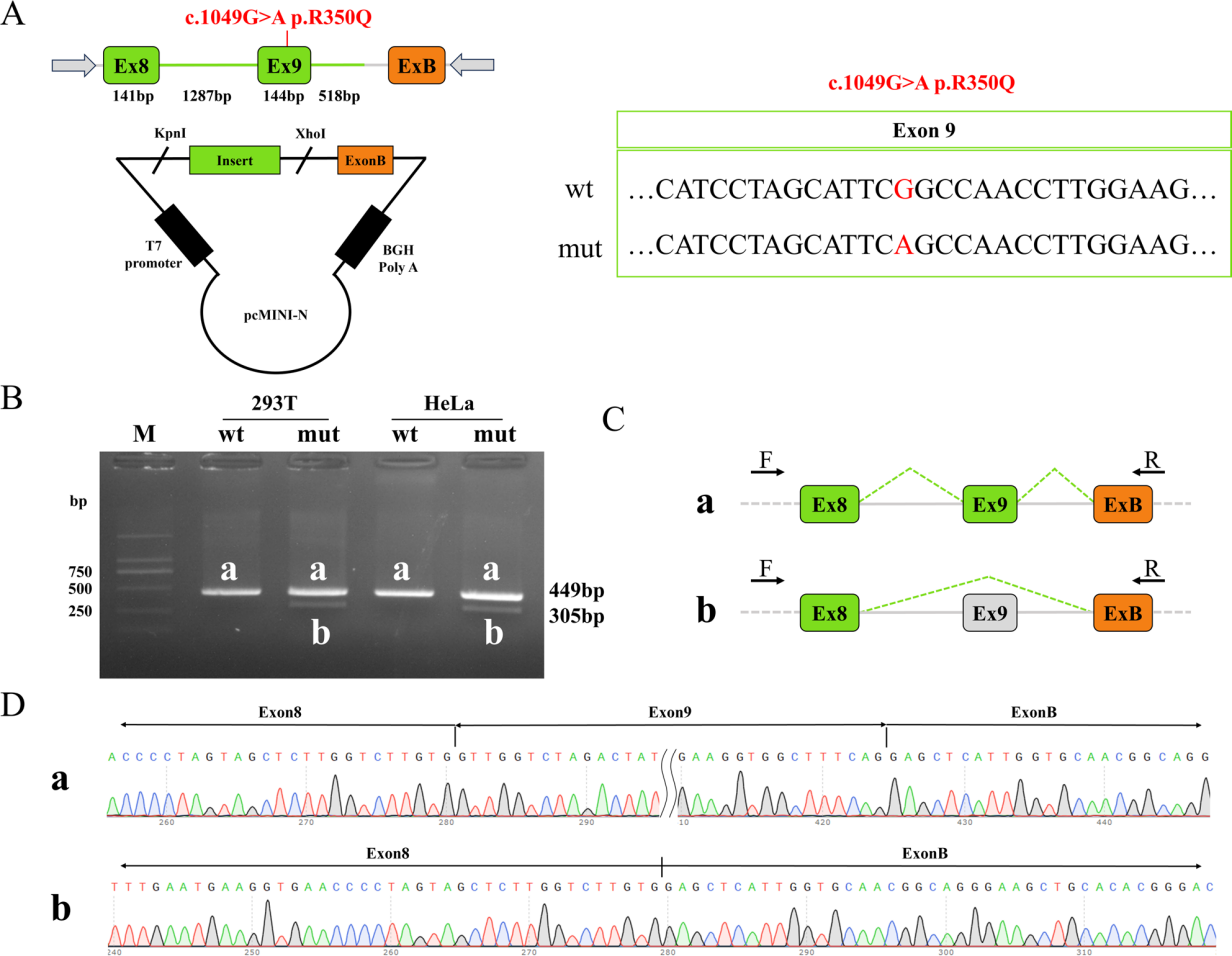
Minigene assay validates splicing outcomes. (**A**) Schematic representation of the minigene construction strategy (primers used are listed in Table 1). (**B**) Agarose gel electrophoresis results from RT-PCR analysis of transcripts; the bands were labeled as a and b in HeLa and 293T cells. (**C**) Diagrammatic depiction of potential minigene splicing outcomes. (**D**) Sequencing traces corresponding to the observed spliced products

To functionally characterize the c.1049G > A variant, we employed a minigene splicing assay containing *ETFDH* exons 8 through 9 (Fig. 4A). RT-PCR analysis of transcripts from transfected HeLa and HEK293T cells revealed distinct splicing patterns between genotypes. While the wild-type construct yielded a single canonical product (~ 449 bp), the mutant construct produced two bands: the full-length wild-type transcript (band a) and a shorter isoform (band b, ~ 305 bp) (Fig. 4B). Schematic analysis indicates that the size difference corresponds to the complete skipping of the 144-bp exon 9 (Fig. 4C). This was confirmed by Sanger sequencing, which showed that band b represents an in-frame deletion resulting from the direct ligation of exon 8 to exon B (Fig. 4D). These results recapitulate the aberrant splicing observed in patient-derived iPSCs, confirming that the c.1049G > A variant is sufficient to disrupt canonical splicing.

### Structural consequences of exon 9 skipping

**Fig. 5:**
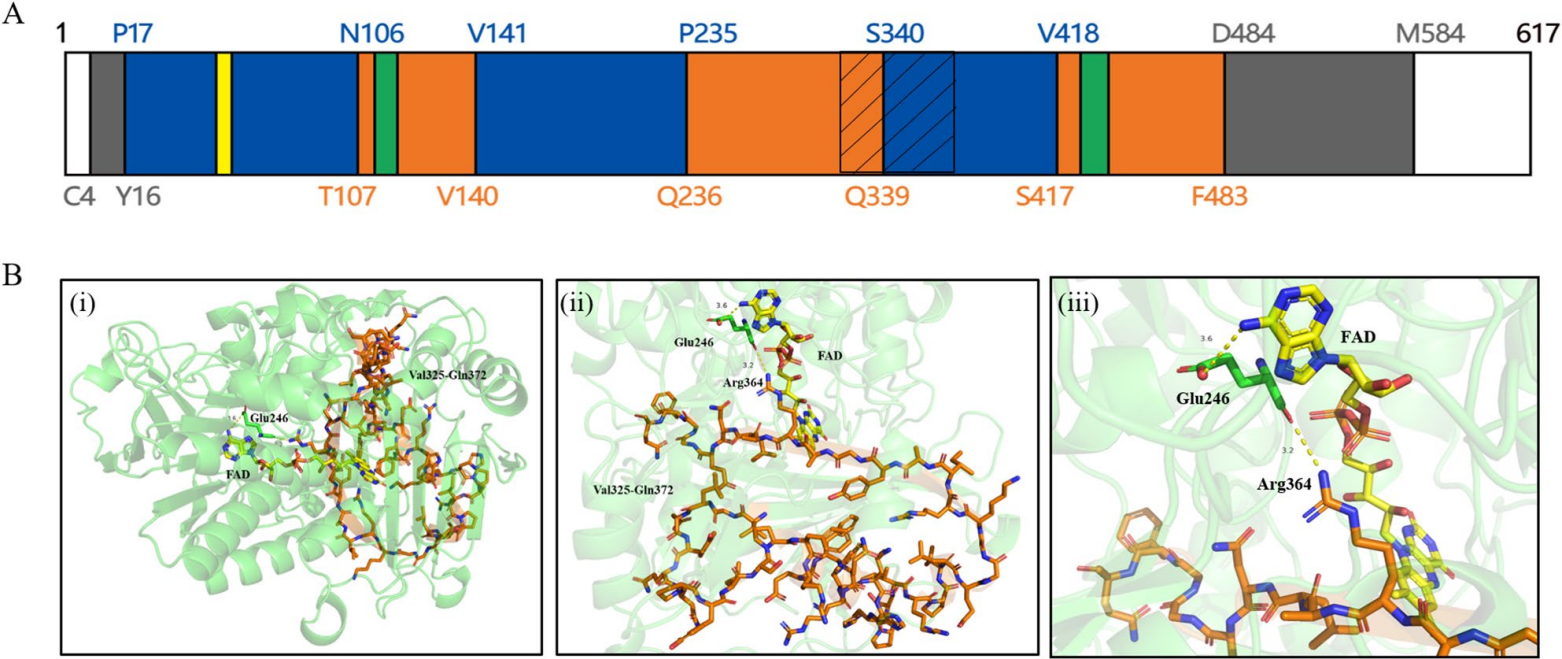
Structural Impact of the Exon 9-Skipping Variant on *ETFDH*. (**A**) Schematic representation of the ETFDH protein domains. The region corresponding to exon 9 (residues Val325-Gln372) is highlighted with hatched lines, indicating its location partially within the FAD-binding domain (blue). Other domains include the 4Fe4S cluster (gray), ADP-binding motif (yellow), UQ-binding domain (orange), and membrane-binding surface (green). (**B**) Structural modeling of the wild-type ETFDH protein in complex with FAD. (i) Overall view of the ETFDH-FAD complex. The deleted region (Val325-Gln372) is highlighted in orange and blue. The FAD cofactor is shown in bright yellow/blue, residue Glu246 in bright green, and the remaining ETFDH protein backbone in pale green. (ii) Zoomed-in view showing the spatial relationship between the Val325-Gln372 segment and the FAD molecule. (iii) Detailed prediction of the interaction network involving Arg364, Glu246, and FAD. Dashed lines indicate hydrogen bonds/salt bridges stabilizing the FAD binding pocket

The confirmed exon 9 skipping results in an in-frame deletion of 48 residues (p.Val325_Gln372del). Domain mapping reveals that this deleted segment constitutes an integral part of the FAD-binding domain (Fig. 5A). To elucidate the mechanistic impact, we generated structural models based on the human ETFDH crystal structure (PDB ID: 2GMJ). In the wild-type conformation, residue Arg364 (located within the deleted segment) forms a critical stabilizing interaction with Glu246, which helps anchor the FAD cofactor (Fig. 5B). Structural modeling indicates that this deletion not only removes a core segment of the FAD-binding domain but also abolishes the stabilizing interaction between Arg364 and Glu246, predicting severely compromised FAD binding affinity and enzymatic activity.

## Discussion

Classically, *ETFDH* genotype-phenotype correlations have been defined by a triad of mutation type, residual enzyme activity, and protein abundance. Severe early-onset MADD (Type I/II) is typically associated with null variants (nonsense, frameshift, or canonical splicing defects) that result in absent ETFDH protein and < 5% residual enzyme activity, often leading to profound metabolic disruption and systemic complications like polycystic kidney disease [10, 18]. In contrast, late-onset forms (Type III) are frequently linked to missense mutations that preserve partial protein stability, yielding 10–30% residual activity and a milder, riboflavin-responsive course [19]. While this binary framework explains many cases, clinical observations of significant phenotypic variability—particularly among patients with non-canonical variants—suggest that these categories represent endpoints of a broader spectrum rather than rigid distinct entities [20–22]. Together with the observed residual protein levels in our case, these molecular defects provide a mechanistic basis for the severe neonatal decompensation followed by clinical and biochemical normalization, distinguishing this trajectory from classic, persistent MADD phenotypes.

The c.1049G > A variant exemplifies how exonic mutations can masquerade as missense changes while primarily disrupting splicing. Unlike canonical defects that abolish protein production, our functional assays confirm c.1049G > A acts as a “leaky” splicing variant, generating a marginal pool of wild-type transcript. This mechanism functions as a dynamic rheostat [12, 20]: the resulting < 10% residual protein was insufficient to meet the high metabolic demands of the neonatal period (triggering decompensation) but proved adequate for basal homeostasis once physiological stressors subsided, enabling spontaneous recovery. While such atypical variants may formally remain classified as VUS in databases, our study clarifies their specific pathogenic mechanism. By defining this critical threshold, we demonstrate how marginal enzyme levels interact with developmental demands to dictate clinical trajectory, providing a functional framework for interpreting similar “gray-zone” variants.

The utility of iPSC technology in modeling inherited metabolic disorders lies in its ability to recapitulate patient-specific genetic contexts within a renewable human cell system [23]. While previous studies have leveraged this platform to validate gene therapy strategies and explore cell-type-specific mechanisms [24, 25], its application to splicing variants offers distinct advantages for disorders like MADD. In our study, patient-derived iPSCs successfully mirrored the aberrant splicing patterns of the c.1049G > A variant observed in minigene assays, confirming that the cellular machinery in human pluripotent cells faithfully processes this “leaky” mutation. However, a limitation of our current work is the assessment at the undifferentiated stage. Given that ETFDH expression varies across tissues, the subtle enzymatic deficits caused by c.1049G > A might be masked in pluripotent cells, precluding direct enzyme activity quantification. Future studies differentiating these iPSCs into specific lineages or organoids would not only enable measurements of mitochondrial respiratory capacity and fatty acid oxidation fluxes under stress [26] but also allow for precise assessment of residual enzyme activity in a tissue-relevant context. Such approaches are essential to delineate the metabolic thresholds underlying the transient phenotype observed in our patient.

In conclusion, this study underscores the utility of iPSCs in functionally characterizing VUS within complex metabolic contexts. We demonstrate that the c.1049G > A variant, although located in an exonic region, acts primarily by disrupting pre-mRNA splicing rather than altering the amino acid sequence directly. This “leaky” splicing defect results in a partial, stress-dependent loss of ETFDH function, providing a mechanistic explanation for the patient’s transient neonatal decompensation followed by recovery. By elucidating how such pseudo-missense variants modulate residual enzyme activity, our findings refine the genotype-phenotype correlation for MADD. Furthermore, this functional evidence offers a more nuanced basis for genetic counseling, highlighting the critical role of environmental triggers in disease manifestation and aiding in the interpretation of similar “gray-zone” variants.

## Supplementary Information

Below is the link to the electronic supplementary material.

Supplementary Material 1

Supplementary Material 2

Supplementary Material 3

Supplementary Material 4

Supplementary Material 5

## Data Availability

The raw sequence data reported in this paper have been deposited in the Genome Sequence Archive (Genomics, Proteomics & Bioinformatics 2021) in National Genomics Data Center (Nucleic Acids Res 2022), China National Center for Bioinformation/Beijing Institute of Genomics, Chinese Academy of Sciences (GSA-Human: HRA007961) that are publicly accessible at https://ngdc.cncb.ac.cn/gsa-human.
