# Supplementary figures and images for: Splicing defect and functional characterization of the ETFDH c.1049G > A VUS underlying transient MADD: an iPSC and minigene study
Source: Orphanet J Rare Dis. 2026 May 29;21:257. doi: 10.1186/s13023-026-04407-1 (PMC13420856; doi:10.1186/s13023-026-04407-1)

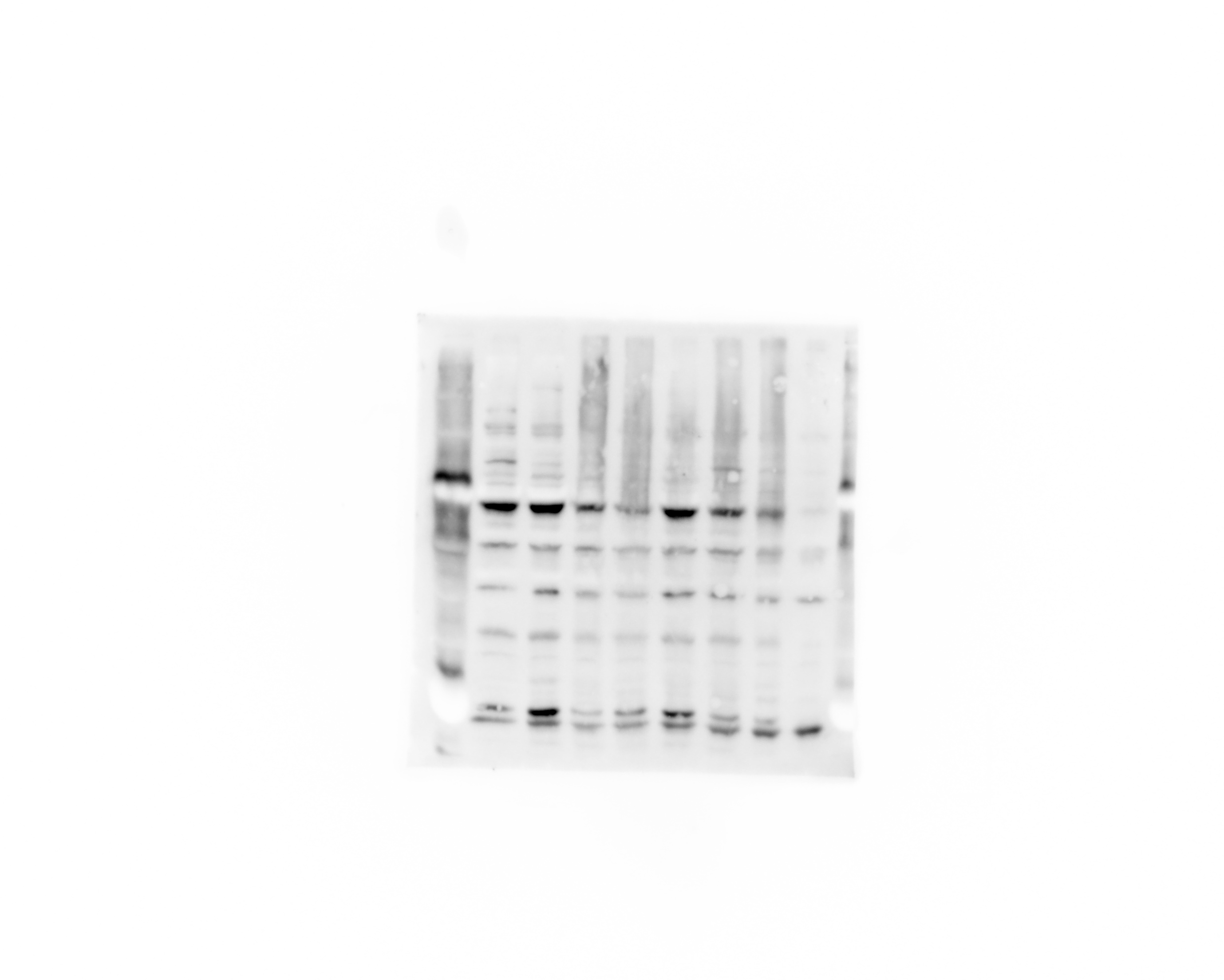

Supplement: Supplementary file 1 — Supplementary Material 1 [file 13023_2026_4407_MOESM1_ESM.jpg]

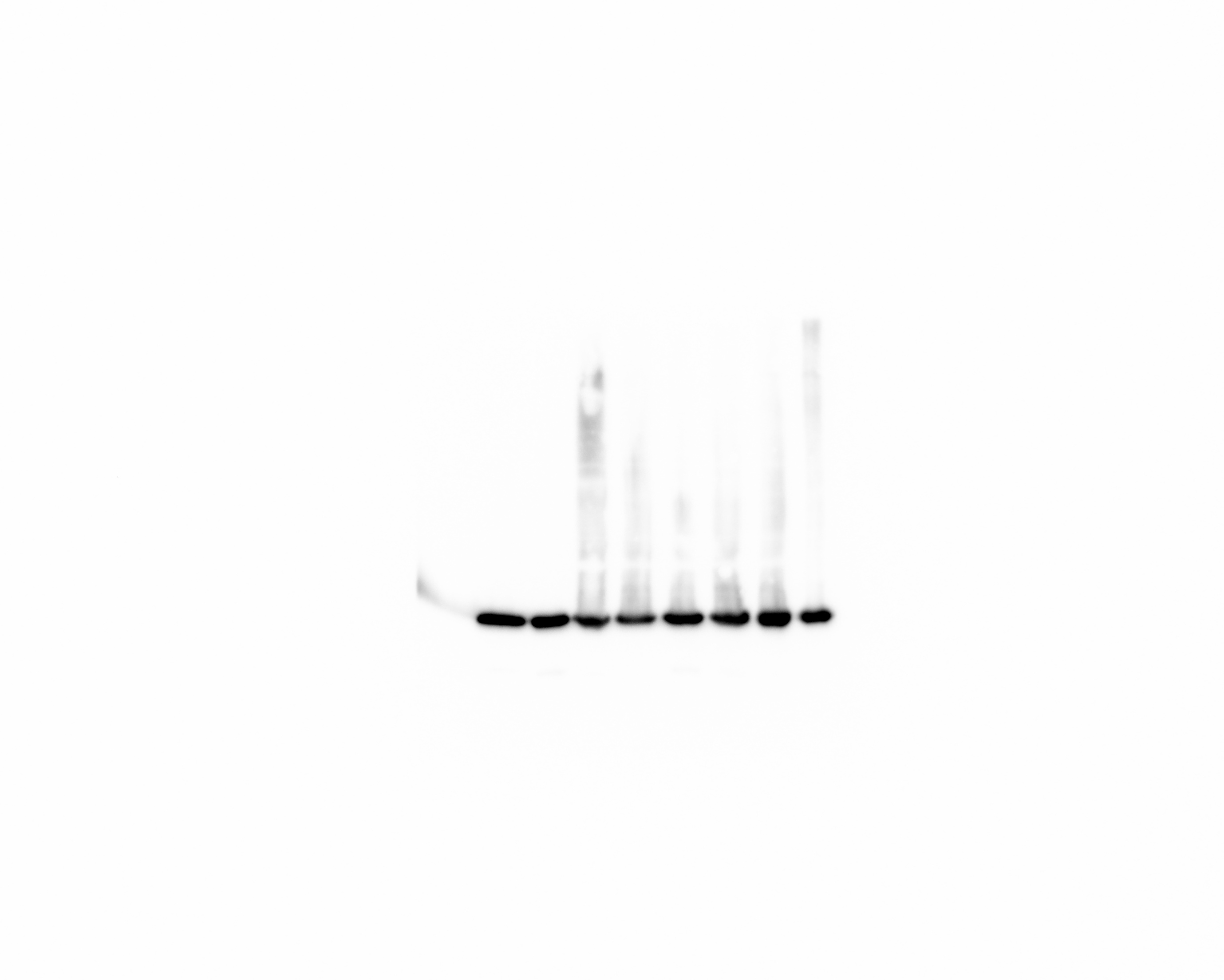

Supplement: Supplementary file 2 — Supplementary Material 2 [file 13023_2026_4407_MOESM2_ESM.jpg]

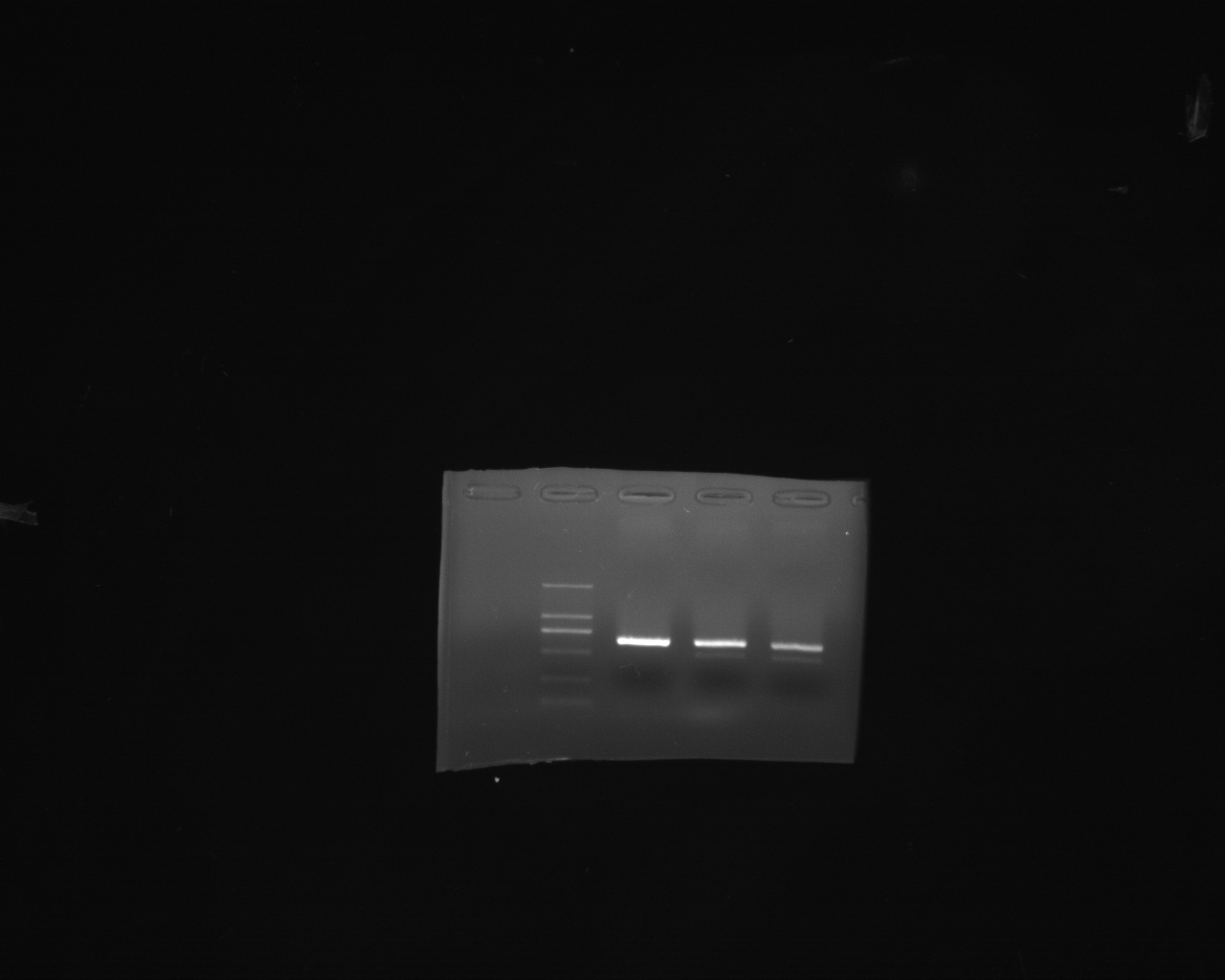

Supplement: Supplementary file 3 — Supplementary Material 3 [file 13023_2026_4407_MOESM3_ESM.tif]

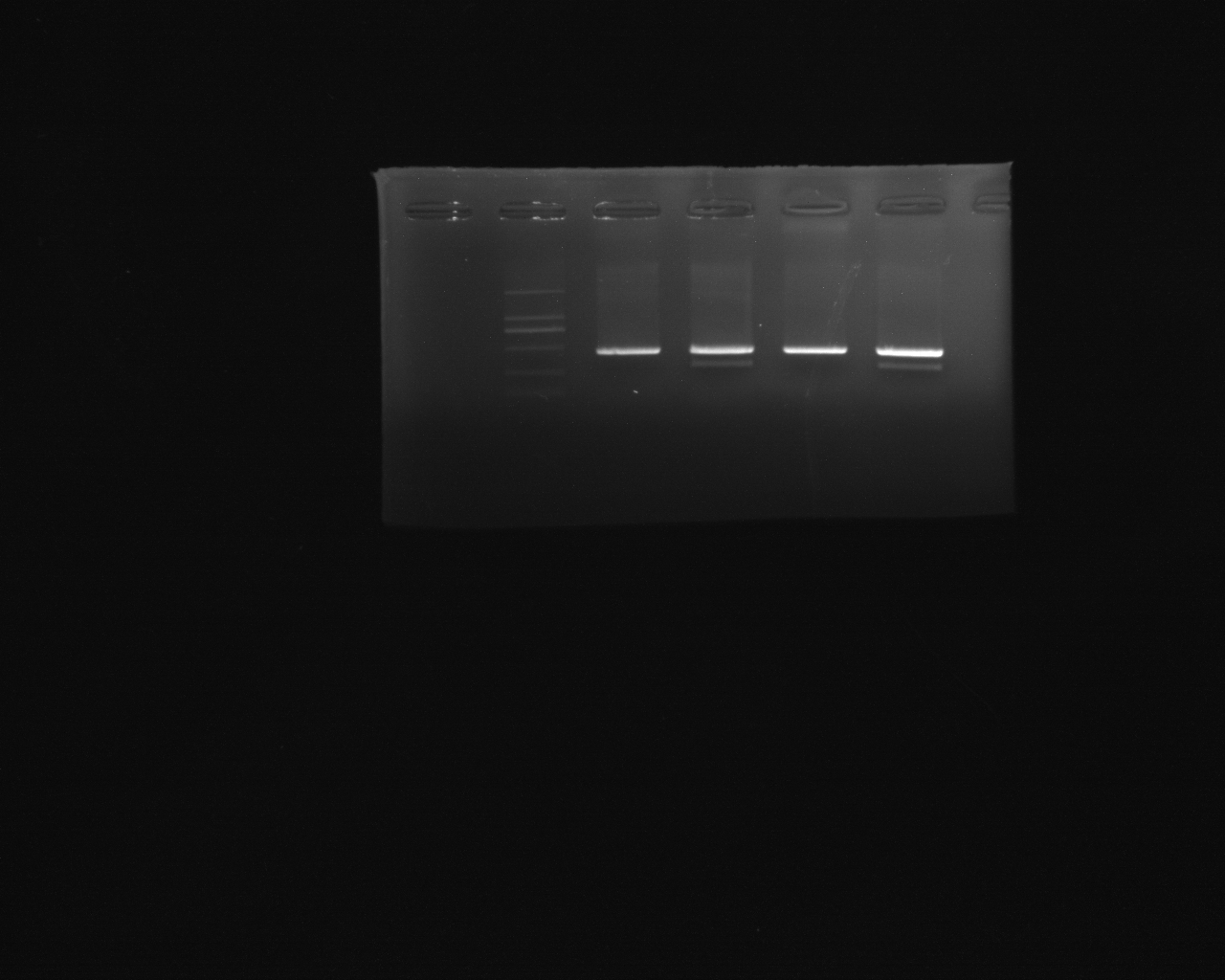

Supplement: Supplementary file 4 — Supplementary Material 4 [file 13023_2026_4407_MOESM4_ESM.tif]
