# Supplementary material for: Splicing defect and functional characterization of the ETFDH c.1049G > A VUS underlying transient MADD: an iPSC and minigene study
Source: Orphanet J Rare Dis. 2026 May 29;21:257. doi: 10.1186/s13023-026-04407-1 (PMC13420856; doi:10.1186/s13023-026-04407-1)
Supplement: Supplementary file 5 — Supplementary Material 5 [file 13023_2026_4407_MOESM5_ESM.doc]

Table S1. Primer Sequences for Sanger Sequencing, Splicing Assays, and Minigene Experiments.

| Primer name | Primer-F (5’ to 3’) | Primer-R (5’ to 3’) |  |
| --- | --- | --- | --- |
| 1049 | TGAGTTCCAGAGCACAAGGAT | GTCACACAATACTTCAATTACACCAT |  |
| 1227 | AGCCTTTCCCTACAGCTCTAG | ATGCAAAGAAAACTACATGGCTTATTC |  |
| E1 | ACAAAAGGATGGTGCACCAA | GCTTGAGCCGTTCAAAGTCA |  |
| E2 | GGACATCTAGCCAAGCAACT | CGTGGCAGGACGGTCTTATA |  |
| A | GTGCAGTGGCATGATCTTGG | GGTACAAGCTTTAGAGCACA |  |
| B | CAGCCTCCCAAAGTGCTAGG | GGTACAAGCTTTAGAGCACA |  |
| pcMINI | GCTTGGTACCatgTTATGGGTTAT  TGATGAAAA | TTTCCTCGAGCTGGTTAAAACAAC  TAACAA |  |
| MT | ACCATCCTAGCATTCaGCCAACC  TTGGAAGG | CCTTCCAAGGTTGGCtGAATGCTAG  GATGGT |  |
| N | CTAGAGAACCCACTGCTTAC | GCCCTCTAGACTGGTCATTCCGGCTC |  |

Table S2. Primer Sequences Utilized for iPSC Induction Verification.

| Gene | Primer-F (5’ to 3’) | Primer-R (5’ to 3’) |
| --- | --- | --- |
| GAPDH | GTGGACCTGACCTGCCGTCT | GGAGGAGTGGGTGTCGCTGT |
| EBNA1 | TTTAATACGATTGAGGGCGTCT | GGTTTTGAAGGATGCGATTAAG |
| Wpre | CCTGCTTCTCGCTTCTGTTC | AAGCCATACGGGAAGCAATA |
| OCT4 | CCTCACTTCACTGCACTGTA | AAGCCATACGGGAAGCAATA |
| SOX2 | CCCAGCAGACTTCACATGT | CCTCCCATTTCCCTCGTTTT |
| NANOG | TGAACCTCAGCTACAAACAG | TGGTGGTAGGAAGAGTAAAG |
| NR2F2 | GACCAGCACCATCGCAACC | GCGCAACAGCAGGGAAAT |
| PAX6 | GTCCATCTTTGCTTGGGAAA | TAGCCAGGTTGCGAAGAACT |
| HAND1 | TCAAGGCTGAACTCAAGAAGG | TGCGTCCTTTAATCCTCTTCTC |
| GATA4 | CGCCCGACACCCCAATCTC | CCGTCCCATCTCGCCTCCA |
| FOXA2 | GGAGCGGTGAAGATGGAA | TACGTGTTCATGCCGTTCAT |
| SOX17 | CTCTGCCTCCTCCACGAA | CAGAATCCAGACCTGCACAA |

Table S3 Clinical characteristics of the subject.

| Subject |  |  |
| --- | --- | --- |
| Gender | Female |  |
| Age at onset | 1D |  |
| Complications during pregnancy | Threatened preterm birth | |
| Weeks’ gestation at delivery | 35+1 |  |
| Birth weight (kg), Apgar score | 2.43, 9 |  |
| Clinical course | Glutaric Aciduria Type Ⅲ | |
| Clinical features |  |  |
| Dyspnea | + |  |
| Acidosis | + |  |
| Hypotonia | + |  |
| Hypoglycemia | + |  |
| Shock | + |  |
| Neonatal Cardiac Dysfunction | + |  |
| Sepsis | + |  |
| Pneumothorax | + |  |
| Poor feeding | + |  |
| Cranial | Intracranial Hemorrhage with Hydrocephalus | |
| Arterial blood gas analysis |  |  |
| PH (reference, 7.35-7.45) | 7.275↓ |  |
| PO2 (reference, 83-108 mm Hg) | 18.2↓ |  |
| PCO2(reference, 35-45 mm Hg) | 52.1↑ |  |
| HCO3- (reference, 22-26 mmol/L) | 24.2 |  |
| BE (reference, -3to +3) | -2.6 |  |
| Blood routine test |  |  |
| White blood cells (reference, 3.5-9.5x10^9^/L) | 14.42 |  |
| Platelet count (reference, 125-350x10^9^/L) | 110 |  |
| Hemoglobin (reference, 180-190 g/L) | 157 |  |
| Blood biochemical tests |  |  |
| ALT (reference, 0-38U/L) | 17 |  |
| Lactic acid (reference, 0.5 -1.6 mmol/L) | 6.8↑ |  |
| Glucose (reference, 3.6-6.1 mmol/L) | 3.4↓ |  |
| Potassium (reference, 3.6-5.2 mmol/L) | 3.36↓ |  |
| Sodium (reference, 135-145 mmol/L) | 146↑ |  |
| Calcium (reference, 2.2-3 mmol/L) | 1.59↓ |  |
| Gamma-glutamyltransferase (reference, 5-49 U/L) | 88.5↑ |  |
| Direct Bilirubin (reference, 0-7 umol/L) | 8.5↑ |  |
| Indirect Bilirubin (reference, 2-15 umol/L) | 157.3↑ |  |
| ammonia (reference, 18-72 umol/L) | 54 |  |
| C reactive protein (reference, 0-10 mg/L) | 77.57↑ |  |
| PCT (reference, ≤0.5 ng/ml) | 22.6↑ |  |
| Cardiac markers |  |  |
| AST (reference, 0-38 U/L) | 113↑ |  |
| CK (reference, 21-220 U/L) | 373↑ |  |
| CK-MB (reference, 0-5 U/L) | 4.27 |  |
| Lactate dehydrogenase (reference, 114-240 U/L) | 1701↑ |  |
| Alpha-hydroxybutyrate dehydrogenase (reference, 74-200 U/L) | 1121↑ |  |
| Coagulation profiles |  |  |
| Prothrombin Time (reference, 10-15 s) | 18.6↑ |  |
| Activated Partial Thromboplastin Time (reference, 24-38 s) | 30.9 |  |
| Thrombin Time (reference, 14-21 s) | 18.3 |  |
| Fibrinogen (reference, 2-4 g/L) | 2.39 |  |
| D-dimer (reference, 0-1 mg/L) | 13.12↑ | |
